# Antifungal Activity of 8-Hydroxyquinoline Derivatives Against *Candida auris*, *Candida haemulonii*, *Cryptococcus neoformans*, and *Cryptococcus gattii* Complex

**DOI:** 10.3390/pathogens14100999

**Published:** 2025-10-02

**Authors:** Maria Eduarda Krummenauer, Matheus da Silva Camargo, Caroline de Bem Gentz, Marcela Silva Lopes, Letícia Feliciani da Luz, Danielle da Silva Trentin, Belisa Ávila Rodrigues, Karine Rigon Zimmer, Saulo Fernandes de Andrade, Marilene Henning Vainstein

**Affiliations:** 1 Centro de Biotecnologia, Universidade Federal do Rio Grande do Sul, Av. Bento Gonçalves, Porto Alegre 91501-970, RS, Brazil; mekrummenauer@gmail.com (M.E.K.); mtsc.camargo@gmail.com (M.d.S.C.); 2 Programa de Pós-Graduação em Biologia Celular e Molecular, Centro de Biotecnologia, Universidade Federal do Rio Grande do Sul—UFRGS, Porto Alegre 91500-970, RS, Brazil; saulo.fernandes@ufrgs.br; 3 Faculdade de Farmácia, Universidade Federal do Rio Grande do Sul, Porto Alegre 90610-000, RS, Brazil; carolgentz@gmail.com (C.d.B.G.); marcela.lopes@ufrgs.br (M.S.L.); 4 Programa de Pós-Graduação em Biociências, Universidade Federal de Ciências da Saúde de Porto Alegre—UFCSPA, Porto Alegre 90050-170, RS, Brazil; leticiafelicianidaluz@gmail.com (L.F.d.L.); daniellest@ufcspa.edu.br (D.d.S.T.); belisavro@gmail.com (B.Á.R.); karinerz@ufcspa.edu.br (K.R.Z.)

**Keywords:** *Cryptococcus* spp., *C. auris*, *C. haemulonii*, 8-hydroxyquinoline, antifungals

## Abstract

Invasive fungal infections and the emergence of antifungal resistance pose significant challenges to public health. This study evaluates the antifungal activity of two 8-hydroxyquinoline derivatives, PH265 and PH276, against *Cryptococcus* spp., *Candida auris*, and *Candida haemulonii*. Using the EUCAST protocol, both compounds demonstrated broad-spectrum antifungal activity, with MICs ranging from 0.5 to 8 μg/mL. PH276 exhibited synergistic effects with fluconazole and caspofungin against *C. haemulonii* (FIC ≤ 0.5). The derivatives inhibited *C. neoformans* biofilm formation at higher concentrations and modulated polysaccharide capsule formation in *Cryptococcus* spp. In vivo toxicity assays in *Tenebrio molitor*, *Galleria mellonella*, and *Caenorhabditis elegans* revealed no significant adverse effects, with survival rates comparable to controls. These findings highlight PH265 and PH276 as promising antifungal agents with biofilm-disrupting properties, capsule-modulating effects, and low toxicity, supporting their potential for therapeutic development.

## 1. Introduction

The escalating prevalence of invasive fungal infections, characterized by increased frequency and resistance to treatment, poses a growing public health concern [1]. It is estimated that fungal diseases kill more than 1 million individuals annually, with therapeutic alternatives being characterized by either toxicity or unaffordability, in regions where neglected populations are more prevalent [2].

*Candida auris*, an emerging fungus characterized by multidrug resistance, is rapidly disseminating globally [3]. Phylogenetically affiliated with *Candida haemulonii*, a fungus renowned for its intrinsic resistance to amphotericin B and fluconazole, *C. auris* infections are intricately associated with heightened mortality rates [4]. The emergence of pan-resistant strains of *C. auris* in certain regions serves as a concerning indicator for a disease characterized by limited treatment options, elevated mortality rates, and the pathogen’s propensity for facile transmission within healthcare settings [5]. It has been proposed that *C. auris* is the first example of a human pathogen that emerged from global warming effects on wetlands [6].

The challenge of antifungal resistance extends beyond *C. auris*; *Cryptococcus* spp. has been exhaustively documented with resistance issues [7,8]. *Cryptococcus neoformans* is an encapsulated yeast responsible for more than 200,000 yearly deaths [9]. *C. neoformans* can also grow as a biofilm, a phenomenon previously associated with increased resistance to antifungal therapies and host immune mechanisms [10]. The standard therapeutic approach for cryptococcosis combines amphotericin B with 5-flucytosine [11]. The fatality of cryptococcosis is associated with poor and late diagnosis, drug resistance, and the limited availability of treatment [12]. The drug 5-fluorocytosine is not available in emerging countries [11], and intravenous treatment requires medical infrastructure, while amphotericin B is nephrotoxic [13]. As an alternative, fluconazole is frequently used, although emerging resistance has been previously described with different strains of *Cryptococcus* spp. and *Candida* spp. [14,15].

The pace of knowledge generation in antifungals is significantly slower compared to antibacterial drug development [16]. This discrepancy directly results in fewer classes of antifungal drugs compared to antibacterial drugs. The latest class of antifungals approved for clinical use was echinocandins in 2002, which were ineffective against *C. neoformans* [17]. Taken together, the rising burden of invasive mycoses and the scarcity of novel antifungal classes highlight the urgent need for alternative scaffolds. In this context, 8-hydroxyquinoline (8HQ) derivatives have attracted attention because of their broad spectrum of biological activities, including antiparasitic and anticancer effects [18,19,20,21], as well as antimicrobial activity [22,23,24,25,26]. Their chemical versatility makes them promising scaffolds for antifungal drug discovery. The derivatives investigated in this study combine the 8HQ scaffold with triazole moieties, two structural features with recognized bioactivity. While triazoles are a well-established class of antifungals, the hybridization of these pharmacophores may provide additive or novel mechanisms of activity, supporting their evaluation against multidrug-resistant fungi.

**Figure 1:**
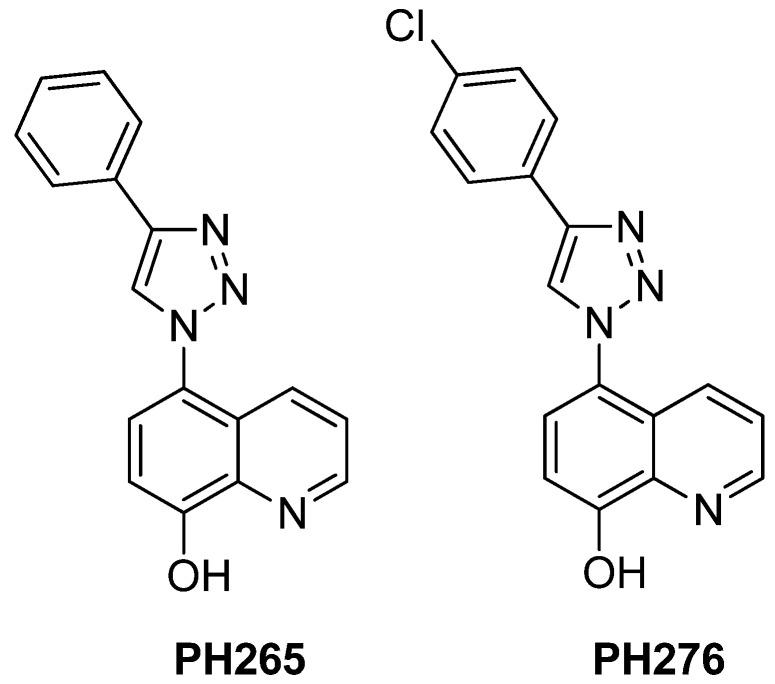
Chemical Structure of 8-hydroxyquinoline Derivatives PH265 and PH276.

To add to the efforts of drug discovery to control fungal infections, in this research, we explored two derivatives of 8HQ—PH265 and PH276 (Figure 1)—to assess their antifungal activity against *Cryptococcus* spp., *C. auris*, and *C. haemulonii*.

## 2. Materials and Methods

### 2.1. Compounds

The 8-hydroxyquinoline derivatives PH265 [27] and PH276 [28] were previously synthesized and fully characterized, as reported in the cited references.

The molecules were diluted to a stock solution of 6000 µg/mL in DMSO and stored at −20 °C until use. Intermediate dilutions were prepared in MilliQ water, ensuring a final DMSO concentration of less than 0.1%. The molecules were serially diluted for the experiments to concentrations ranging from 32 to 1 µg/mL, prepared as two-fold concentrated solutions.

### 2.2. Strains and Growth Conditions

*Cryptococcus neoformans* strain H99, *Cryptococcus deneoformans* strain B3501, and *Cryptococcus deuterogattii* strain R265 were maintained on yeast extract peptone dextrose (YPD) agar. *Candida auris* strain Ca446 and *Candida haemulonii* strain LipCh12 were maintained on Sabouraud (SB) agar. For liquid culture, strains were grown in YPD or SB broth (as appropriate) at 30 °C under agitation (180 rpm) overnight. Prior to the assays, cells were washed three times with phosphate-buffered saline (PBS).

*C. elegans* N2 Bristol (wild-type) was maintained in Petri dishes containing Nematode Growth Medium (NGM: NaCl, peptone, CaCl_2_, cholesterol, MgSO_4_, phosphate buffer, nystatin, streptomycin, and agar) seeded with *Escherichia coli* OP50 at 20 °C [29]. Before the tests, worms were synchronized using an alkaline hypochlorite solution (“bleaching”) to obtain worms at the same larval stage [30].

The lifecycle of *Galleria mellonella* was maintained under controlled laboratory conditions. Moths, eggs, and larvae were kept at temperatures of 22 °C, 28 °C, and 32 °C, respectively. The larvae were fed a laboratory diet consisting of wheat flour (14.7%), coarse wheat bran (14.7%), wheat germ (14.7%), powdered milk (29.4%), liquid honey (8.8%), glycerol (8.8%), and brown sugar (8.8%), until they reach the weight to be used in the assays.

### 2.3. Analysis of Antifungal Activity

The values of Minimum Inhibitory Concentrations (MICs) were determined using the protocol proposed by the European Committee on Antimicrobial Susceptibility Testing (EUCAST) with minor modifications. Molecules and commercial antifungal agents were serially diluted in two-fold steps within the range of 32 to 0.125 µg/mL; for fluconazole, the range was extended up to 64 µg/mL. Dilutions were prepared as two-fold concentrated solutions in 50 µL of sterile MilliQ water and incubated with 50 µL of fungal suspensions containing 5 × 10^5^ cells/mL in RPMI 1640 medium (Thermo Fisher Scientific, Waltham, MA, USA) (two-fold concentrated, pH 7.2, 2% glucose) buffered with 3-(*N*-morpholino)propanesulfonic acid (MOPS) in 96-well plates. The plates containing cryptococcal cells (H99, B3501, and R265) were incubated at 37 °C for 48 h, and the plates containing *C. auris* and *C. haemulonii* strains (Ca446 and LipCh12) were incubated at 30 °C for 24 h. The optical density at 530 nm was determined spectrophotometrically, and the MIC was defined as the minimum concentration that inhibited > 50% of fungal growth (PH 265, PH 276, fluconazole, and caspofungin) or inhibited > 90% of fungal growth (amphotericin B) when compared to the control without treatment.

To determine the Minimum Fungicidal Concentration (MFC), 5 µL of each well from the MICs plate was transferred to SB agar and incubated for 24 h at 30 °C for *C. auris* and *C. haemulonii* or incubated on YPD agar for 48 h at 37 °C for cryptococcal cells [16]. All experiments were performed in triplicate on at least three independent biological replicates. The detection limits for MIC and MFC determinations corresponded to the concentration ranges described above for each molecule (0.125–32 µg/mL for all compounds and antifungals, except for fluconazole, which was tested up to 64 µg/mL).

### 2.4. Inhibition of Biofilm Formation by 8-Hydroxyquinoline Derivatives

A semiquantitative measurement of *C. neoformans* biofilm formation was performed using the XTT [2,3-bis-(2-methoxy-4-nitro-5-sulfophenyl)-2H-tetrazolium-5-carboxanilide] reduction assay with an electron-coupling reagent. XTT is a tetrazolium salt that is reduced by mitochondrial enzymes of metabolically active cells to a water-soluble orange formazan product, and it is widely used in colorimetric assays to evaluate cell metabolic activity. Fungal cells were grown overnight in YPD broth, washed twice with PBS, and adjusted to a concentration of 2 × 10^7^ cells/mL. Fungal suspensions (50 µL) were mixed with 50 µL of each test molecule, serially diluted (32 to 1 µg/mL, two-fold concentrated), in RPMI 1640 medium in a 96-well microtiter plate. The plates were incubated for 48 h at 37 °C to allow biofilm formation.

After incubation, wells were washed twice with PBS to remove non-adherent cells. For biofilm quantification, 50 µL of XTT salt solution (0.3 mg/mL) with the electron-coupling reagent was added to each well, followed by incubation at 37 °C for 5 h. Mitochondrial dehydrogenase activity in metabolically active yeast cells within the biofilm reduced the XTT tetrazolium salt to XTT formazan, resulting in a colorimetric change. The absorbance was measured at 492 nm using the Thermo Fisher Scientific Varioskan LUX (Thermo Fisher Scientific, Waltham, MA, USA).

### 2.5. Evaluation of Cryptococcal Main Virulence Factors

The urease activity, capsule formation, and pigmentation of *Cryptococcus* spp. were evaluated when the yeast was treated with the PH265 and PH276 MIC and MIC × 0.5 [31].

For capsule size evaluation, cryptococcal cells (2 × 10^6^ cells/mL) were incubated in RPMI 1640 (two times concentrated, pH 7.2; 2% glucose) buffered with MOPS containing the molecules concentrations for 72 h at 37 °C under a 5% CO_2_ atmosphere (≥99.5% purity), supplied by Oxi Products and used in all experiments [32].

Melanin production was visually analyzed by plating 10^5^ fungal cells in the minimal medium agar with L-dopa (1 mM) and incubating with the molecule for 72 h at 37 °C under a 5% CO_2_ atmosphere.

Urease activity was evaluated using the methods proposed by [33] with minor modifications. Cryptococcal cells (10^8^ cells/mL) and the active molecules were incubated in rapid Robert’s urea broth (RUH) for 4 h at 37 °C in a rotatory shaker at 700 rpm. The fungal cells were centrifuged for 3 min at 5000 rpm. The supernatant was collected and diluted with MilliQ water. The absorbance at 560 nm (OD_560_) was read. All readings above 0.3 indicated ureolysis.

### 2.6. Analysis of Synergistic Effects

The synergistic activity between PH265 and PH276 and standard drugs was determined based on the calculation of the Fractional Inhibitory Concentration Index (FIC) [34]. The molecules analyzed in this study (denominated drug A) were serially diluted (8 to 0.0625 µg/mL) in 96-well plates. Standards of antifungals (denominated drug B) were serially diluted (8 dilutions) from 2 to 0.003 µg/mL (amphotericin B), 64 to 0.0625 µg/mL (fluconazole), or 8 to 0.015 µg/mL (caspofungin) [35]. FIC was defined as: $\frac{MIC\;drug\;A\;combined}{MIC\;drug\;A\;alone}+\frac{MIC\;drug\;B\;combined}{MIC\;drug\;B\;alone}$. Synergism was categorized as follows: Synergistic effect, FIC < 0.5; no effect, FIC < 0.5–4; antagonist effect, FIC > 4 [34].

### 2.7. Confocal Microscopy

To evaluate the cryptococcal capsule, confocal laser scanning microscopy was performed Olympus FluoView 1000 confocal laser scanning microscope at the Universidade Federal do Rio Grande do Sul (Olympus Corporation, Tokyo, Japan). The capsule induction was prepared as described above. Cells were fixed with 4% paraformaldehyde in PBS for 1 h min at room temperature. No permeabilization step was performed. The fluorescent microscopy was performed as described by [36] with minor modifications. Cells were washed with PBS and incubated with 200 μL of Wheat Germ Agglutinin (WGA) conjugated to Alexa 488 (Thermo Fisher Scientific, Waltham, MA, USA) (5 μg/mL, green) for 30 min at 37 °C under agitation at 600 rpm. After washing with PBS, cells were incubated with 200 μL of Calcofluor White (Biotium, Inc., Santa Clara, CA, USA) (5 μg/mL, blue) for 30 min at 37 °C under agitation at 600 rpm. Subsequently, cells were incubated with 200 μL of 18B7 antibody (10 μg/mL) for 30 min at 37 °C under agitation at 600 rpm, followed by incubation with antigoat IgG conjugated to Alexa 638 (200 μL, 1000 μg/mL, red) for 30 min at 37 °C under agitation at 600 rpm. All staining steps were performed in the dark to protect the fluorophores. After each incubation, cells were washed three times with PBS to remove excess reagent. Finally, the fungal cells were resuspended in PBS and examined by confocal microscopy.

### 2.8. Toxicity

#### 2.8.1. Toxicity in *Tenebrio molitor*

*Tenebrio molitor* larvae were obtained from a local supplier and reared on an oatmeal-based diet until used for experimentation. The assay was conducted following [37], with minor modifications. To assess the toxicity of PH265 and PH276, 5 µL of each compound was injected into the ventral side of the larvae at the second segment behind the legs using a Syringe (PerkinElmer, Waltham, MA, USA). Twenty larvae of similar size (approximately 110 mg) were selected for each group. The concentrations tested corresponded to the average MICs determined (2.2, 8.8, 1.1, and 35.2 µg/g larvae for PH265, PH276, amphotericin B, and fluconazole, respectively) and the maximum concentration tested for biofilm inhibition (70.4 µg/g larvae). Controls consisted of larvae injected with MilliQ water or DMSO alone. The larvae were maintained in Petri dishes at 28 °C, and survival was monitored every 24 h for 7 days.

#### 2.8.2. Toxicity in *Galleria mellonella* Larvae

PH265 and PH276 toxicity was evaluated using the *G. mellonella* larvae model. Groups of ten larvae weighing 200–240 mg were used. The larvae received 10 µL of the PH 265 solution at 2.2 or 70.4 mg/kg, and PH276 solution at 8.8 mg/kg, through systemic injection into the hemocoel in the last right proleg using a 10 µL Hamilton Microliter syringe (Hamilton Company, Reno, NV, USA), purchased from Sigma-Aldrich (St. Louis, MO, USA). Controls included a group of larvae inoculated with vehicle (8% DMSO)—negative control for toxicity, and another group of larvae inoculated with 99.5% DMSO (Sigma-Aldrich, USA)—positive control for toxicity. Larvae were assessed daily for survival up to 5 days post-treatment and were evaluated according to survival, being scored as dead when they displayed no movement in response to touch. Experiments were repeated at least 3 times (10 larvae per group).

#### 2.8.3. Toxicity in *Caenorhabditis elegans*

L1 stage *C. elegans* worms were subjected to acute treatment, where 1500 worms were exposed to PH265 (1 µg/mL and 32 µg/mL) or PH276 (4.2 µg/mL and 32 µg/mL) for 30 min. Worms treated with 2% DMSO (Sigma-Aldrich, USA) were used as controls. After treatment, worms were washed three times with 0.5% saline solution to remove residual compounds and then transferred to plates seeded with *E. coli* OP50 as a food source [38].

The survival assay was performed 48 h after treatment. Worm survival was assessed based on movement and responsiveness to mechanical stimuli. A worm was considered dead if it failed to move or respond. The assays were conducted in duplicate and repeated three times independently, with results expressed as a percentage of the control group [38].

Pharyngeal pumping, a reliable indicator of worm health, was evaluated by counting contractions and relaxations of the terminal bulb of the pharynx. Five L4-stage worms at each concentration were individually observed over three 10 s intervals using the EVOS™ FL Auto 2 Imaging System (Thermo Fisher Scientific). The average number of contractions was multiplied by 6 to estimate the total number of pharyngeal pumps per minute. Each assay was performed in duplicate and repeated three times independently, with results expressed as mean and standard deviation [39].

For all in vivo toxicity assays, the concentrations tested were selected to correspond to the average MICs previously determined and, when applicable, to the maximum concentration used for biofilm inhibition. This design allowed us to estimate a preliminary safety index by directly comparing antifungal efficacy (MIC values) with the absence of significant toxic effects in invertebrate models.

### 2.9. Statistical Analyses

For microbiological data, statistical analyses were performed using GraphPad Prism version 8.0.1 and R (version 4.3.1; R Core Team). To analyze toxicity in the *T. molitor* survival curve, the log-rank test for trend and the Gehan-Breslow-Wilcoxon test was used. Survival curves of toxicity assay in *G. mellonella* larvae were determined using Kaplan–Meier, and statistical significance was determined using the log-rank test. For toxicity assays in *C. elegans*, data were analyzed by One-Way ANOVA, followed by Tukey’s post-test, and a *p*-value ≤ 0.05 was considered statistically significant. For the biofilm inhibition and capsule modulation assays in *Cryptococcus* spp., statistical analyses were performed in R (version 4.3.1) using the readxl, tidyverse, car, FSA, and ggpubr packages. Non-parametric data were analyzed using the Kruskal–Wallis test followed by Dunn’s post hoc test with Bonferroni correction.

## 3. Results

### 3.1. Antimicrobial Activity and Synergistic Potential of 8-Hydroxyquinoline Derivatives

The 8-hydroxyquinoline derivatives investigated in this study demonstrated in vitro antimicrobial activity against all tested fungal isolates, with MIC ranging from 8 to 0.5 μg/mL for PH276 and 1 to 0.5 μg/mL for PH265 (Table 1). The 8HQ compounds demonstrate fungicidal activity against *Cryptococcus* spp. as shown in Table 2. The potential for synergistic effects was evaluated with fluconazole and amphotericin B against *C. neoformans*, and with fluconazole, amphotericin B, and caspofungin against *C. auris* and *C. haemulonii*. The sole combination of molecules that exhibited synergism was the PH276 molecule with caspofungin (Table 3).

### 3.2. PH265 and PH276 Modulate Capsule Formation in Cryptococcus spp.

**Figure 2:**
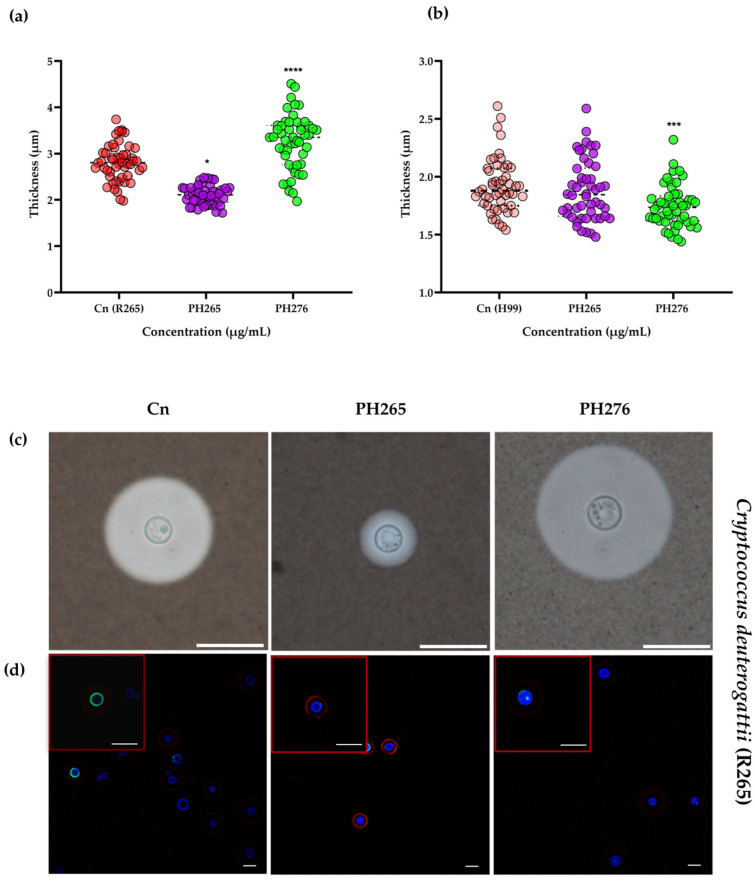
Effects of 8-Hydroxyquinoline Derivatives on the Capsule Formation of *Cryptococcus* spp. (**a**) Measurement of capsule dimensions in *C. deuterogattii* R265 and (**b**) *C. neoformans* H99 following treatment with the 8HQ derivatives PH265 (1 µg/mL) and PH276 (0.5 µg/mL). Capsule size was determined by subtracting the cell body diameter (bounded by the cell wall) from the overall cell diameter, limited by the borders of the capsule. Statistical analysis was performed using Kruskal–Wallis test followed by Dunn’s post hoc test with Bonferroni correction. Significant reductions in capsule size were observed for R265 strain: PH265 (*p* < 0.0001; ****), PH276 (*p* = 0.0154; *) and H99 strain: PH276 (*p* = 0.0003; ***), PH 265 (*p* = 0.0492; *). (**c**) Light microscopy images of *C. deuterogattii* strain R265 cells following cultivation with PH 265 (1 µg/mL) and PH 276 (0.5 µg/mL). Scale bar: 20 µm. (**d**) Confocal microscopic images of *C. deuterogattii* strain R265 cells. The red box highlights a representative cell that is shown at higher magnification to provide a detailed view of capsule morphology. The cells were stained for cell wall chitin (resulting in blue fluorescence from CFW), chitooligomers (resulting in green fluorescence from WGA-Alexa 488), and the capsule (using antigoat IgG conjugated with Alexa 638 resulting in red), as detailed in the Methods section. Scale bar: 20 µm.

*Cryptococcus* spp. virulence factors were assessed, revealing that the PH265 compound exhibited a reduction in polysaccharide capsule formation, whereas the PH276 compound demonstrated an enhancement for the R265 strain. Corroborating the results obtained with Indian ink, alterations in the polysaccharide capsule were visualized using confocal microscopy (Figure 2).

### 3.3. PH265 and PH276 Reduce Cryptococcus Biofilm Formation at High Concentrations

**Figure 3:**
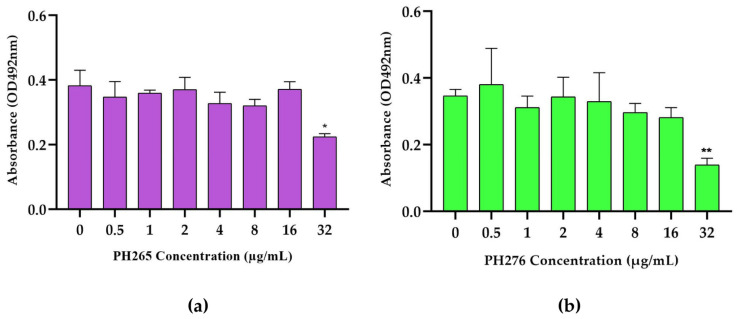
Analysis of Biofilm Inhibition in the Presence of 8HQs Derivative Compounds. (**a**) Biofilm Inhibition Assessed by metabolic activity of untreated and PH265-treated cells or (**b**) PH276-treated *C. deneoformans* strain B3501 biofilms measured by the XTT reduction assay. Statistical comparisons were performed using Kruskal–Wallis test followed by Dunn’s post hoc test with Bonferroni correction. Significant differences were observed between 0 µg/mL and 32 µg/mL for PH265 (*p* = 0.0492; *), and PH276 (*p* = 0.0111; **).

The 8HQ derivatives demonstrated a remarkable ability to inhibit the biofilm formation of *C. deneoformans* at higher concentrations, with effective concentration observed in 32 µg/mL (Figure 3). This inhibitory effect highlights the potential of these compounds as promising agents for disrupting the structural integrity of fungal biofilms, a critical virulence factor that contributes to persistence and resistance in pathogenic fungi.

### 3.4. The Compounds PH265 and PH276 Demonstrated No Significant Toxicity Across All Tested Invertebrate Models

**Figure 4:**
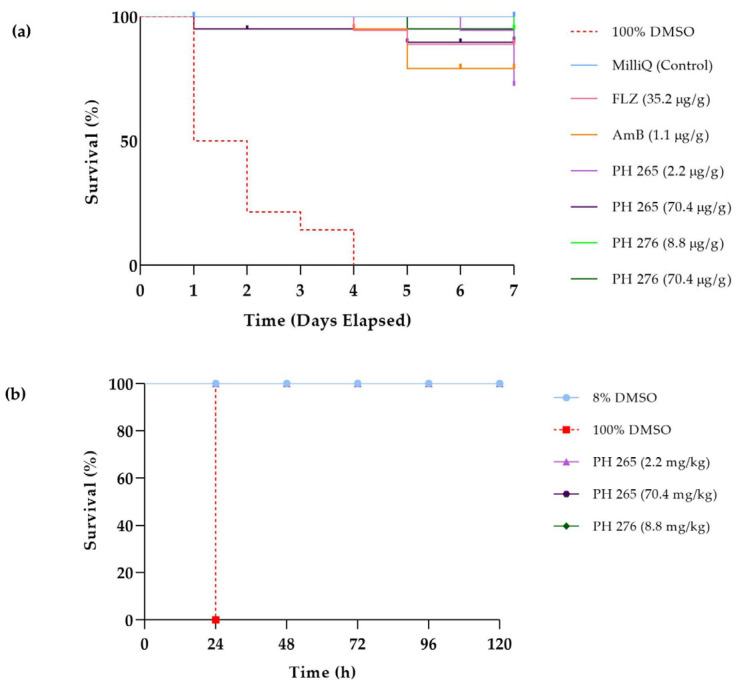
Toxicity Evaluation of PH265 and PH276 in *T. molitor* and *G. mellonella*. (**a**) Survival curve of *T. molitor* for 7 days and (**b**) *G. mellonella* larvae treated with different concentrations of PH265 (2.2 and 70.4 mg/kg) and PH276 (8.8 mg/kg).

**Figure 5:**
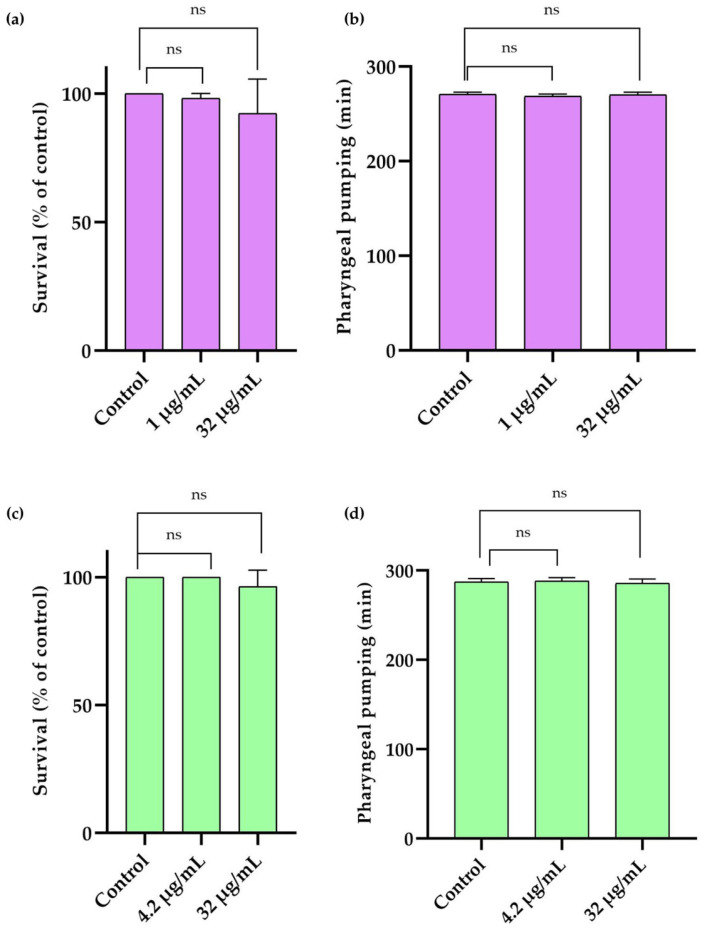
Evaluation of toxicity of PH265 and PH276 in *C. elegans*. (**a**,**c**) Survival rates of worms treated with PH265 and PH276, respectively, compared to the control group (worms treated with the vehicle, DMSO). Data are presented as a percentage of the control group, with no significant differences observed between treatments (One-way ANOVA with Tukey’s post-test). (**b**,**d**) Pharyngeal pumping rates of worms treated with PH265 and PH276, respectively, compared to the control group. Data are presented as mean ± standard deviation, with no significant (ns) differences observed between treatments (One-way ANOVA with Tukey’s post-test).

In *G. mellonella*, all groups treated with PH265 or PH276 showed 100% survival up to 120 h of evaluation, while the positive control (100% DMSO) caused complete host death within the first 24 h (*p* < 0.0001) (Figure 4b). Similarly, in *T. molitor*, the survival curves for PH265 and PH276 closely resembled those of the control and standard treatment groups, indicating tolerance to the tested molecules (Figure 4a). Consistent with these findings, PH265 and PH276 did not impact the survival or pharyngeal pumping of *C. elegans*, further confirming the lack of toxicity in this model (Figure 5). These results collectively support the safety of PH265 and PH276 in invertebrate models.

When comparing the MIC values of PH265 (2.2 µg/mL) and PH276 (8.8 µg/mL) with the highest non-toxic doses in invertebrate models, both compounds showed a favorable preliminary safety index. At concentrations equivalent to or higher than their average MICs, no significant toxic effects were observed in *T. molitor*, *G. mellonella*, or *C. elegans*, supporting their potential therapeutic window.

## 4. Discussion

The therapeutic protocols for treating infections caused by *Cryptococcus* spp., *C. haemulonii*, and *C. auris* are constrained by resistance [7], limited drug availability [40] and toxicity [41]. Some strains are even reported to exhibit multidrug resistance against the three major antifungal classes [42], underscoring the need to explore and develop novel therapeutic alternatives. In this study, we evaluated two synthetic compounds, derivatives of 8HQ: PH265 and PH276. Previous structure–activity relationship studies have shown that 8-HQ derivatives containing a benzene ring separated by a sulfonamide spacer display promising antifungal activity against *Candida* spp. and dermatophytes, reinforcing the potential of 8-hydroxyquinolines as scaffolds for antifungal drug development [43]. Based on these findings, our approach in the present work was to design a non-classical bioisosteric substitution, replacing the sulfonamide spacer with a triazole group. In this context, the triazole functions as a structural linker between the 8HQ and the benzene moieties, without a direct pharmacophoric role, aiming to preserve and potentially enhance antifungal properties.

The MICs of the PH265 molecule did not significantly vary among different strains, with all strains exhibiting a value of 1 µg/mL, except for the *C. deneoformans* B3501 strain, which showed a value of 0.5 µg/mL. In contrast, the PH276 molecule demonstrated higher MICs for *Candida* spp., with 8 µg/mL values for *C. auris* and *C. haemulonii*. In *Cryptococcus* spp., MICs ranged from 0.5 to 4 µg/mL. Neither derivative of the 8HQs showed fungicidal activity for *Candida* spp. at the tested concentrations (MFC > 16 µg/mL). The PH276 molecule exhibited a MIC range of 2 to 16 µg/mL for the tested *Cryptococcus* strains. Additionally, PH276 demonstrated MICs like those found for fluconazole in *C. auris* and *C. haemulonii*, while showing lower MIC values than fluconazole for *Cryptococcus* spp. On the other hand, PH265 exhibited more favorable MICs for *Candida* strains compared to both fluconazole and caspofungin. For *C. haemulonii*, the MIC was at half the concentration found for amphotericin B. In this context, the activity of PH265 and PH276 demonstrates their potential as promising scaffolds for antifungal development. Importantly, their MIC values were comparable or superior to those of reference drugs against resistant *Candida* spp. and *Cryptococcus* spp., suggesting clinical relevance.

To assess whether the association of 8HQ derivatives with amphotericin B, fluconazole, or caspofungin results in improved activity against *C. auris*, *C. hameulonii*, and *C. neoformans*, the FIC was calculated (Table 3). The calculated FIC reveals that the combination of PH276 with caspofungin (FIC = 0.5) or fluconazole (FIC = 0.37) enhances antifungal efficacy, suggesting that these mixtures could reduce the required concentrations of each drug, potentially minimizing toxicity and improving treatment outcomes. This synergy highlights the potential of PH276 as a valuable adjuvant in antifungal therapy, particularly for tackling multidrug-resistant pathogens such as *C. haemulonii*. Such interactions may allow lower effective doses, reducing toxicity and improving treatment outcomes, particularly for *C. haemulonii*, a pathogen for which therapeutic options are extremely scarce. This supports the idea that these derivatives could be explored not only as standalone agents but also as adjuvants to existing antifungals.

Enhanced resistance to antibiotics is a common characteristic linked with biofilm formation. The resistance of *C. neoformans* biofilms to conventional treatments is noteworthy, particularly in the increasing utilization of ventriculoperitoneal shunts to manage intracranial hypertension [10]. PH265 and PH276 at higher concentrations than MIC (>8 µg/mL) show a significant reduction in the biofilm formation of *C. deneoformans.* However, the limited solubility of these molecules poses a challenge, as the DMSO concentration cannot exceed 0.1% in in vitro studies, restricting the ability to test higher concentrations. Addressing this limitation through strategies such as nanoencapsulation [44] could enhance the solubility and bioavailability of the compounds, enabling the exploration of their full therapeutic potential. Since biofilm reduction is often a dose-dependent phenomenon, improving solubility could amplify the efficacy of these 8HQ derivatives in combating biofilms, providing a promising avenue for future research.

The complexities in treating cryptococcosis arise from the virulence determinants inherent to *Cryptococcus* spp. infection. It is known that certain compounds influence key virulence factors; for example, glyphosate and voriconazole inhibit melanization [31,45], EDTA inhibits ureolytic activity in *C. neoformans* [33], mebendazole, dequalinium chloride, bleomycin sulfate and pentamidine isethionate has been described with the ability induced diminished capsules and clioquinol with the ability to caused increase in the capsule size [16,35]. The effects on urease, melanin, and capsule production were tested for the potential of 8HQ derivatives to modulate key virulence factors. It was shown that urease and melanin production remained unaffected by the compounds; however, significant effects were observed on polysaccharide capsule formation. PH265 significantly reduced capsule size, which could impair fungal evasion of host immune defenses and thus enhance therapeutic potential. In contrast, PH276 increased capsule size in the R265 strain. This paradoxical effect may represent an adaptive stress response of the fungal cell. Capsule thickening has been reported under various stress conditions, including antifungal pressure, and may reflect a transient compensatory mechanism prior to growth inhibition. Understanding the dynamics and mechanisms of this response will be important for future studies, as it may help optimize dosing regimens or structural modifications to avoid undesired capsule enhancement.

The survival curve in *T. molitor* demonstrated similar patterns for the PH265 and PH276 molecules compared to both the control group and the standard treatments, indicating an apparent tolerance to the tested compounds. This observation aligns with results from other invertebrate models, such as *G. mellonella*, where all groups treated with PH265 or PH276 showed 100% survival up to 120 h, in stark contrast to the positive control (100% DMSO), which caused complete mortality within the first 24 h (*p* < 0.0001). Additionally, in *C. elegans*, neither compound affected survival or pharyngeal pumping, further reinforcing their safety. Finally, the absence of toxicity in three different invertebrate models reinforces the safety of these compounds, even at concentrations exceeding their MICs. Together, these results suggest that PH265 and PH276 are promising candidates for antifungal therapy, with potential clinical application against multidrug-resistant pathogens. Future studies should include in vivo assays in vertebrate models, pharmacokinetic profiling, and mechanistic investigations to confirm safety and efficacy, paving the way for their development as antifungal agents.

## 5. Conclusions

The 8-hydroxyquinoline derivatives PH265 and PH276 exhibited promising antifungal activity against *Cryptococcus* spp., *C. haemulonii*, and *C. auris*, with low MIC values. PH276 showed synergistic effects when combined with fluconazole or caspofungin against *C. haemulonii*. Both molecules modulated the polysaccharide capsule formation of *Cryptococcus* spp. and significantly reduced biofilm formation at high concentrations. Additionally, they demonstrated the absence of toxicity in invertebrate models, highlighting their potential as candidates for treating the studied fungal pathogens. Future investigations should focus on strategies to enhance compound solubility, as well as comprehensive in vivo studies in mammalian models to evaluate pharmacokinetics, biodistribution, and toxicity. Such studies will be critical to validate the therapeutic efficacy and safety of PH265 and PH276, paving the way for their advancement as novel antifungal agents.

## Figures and Tables

**Table 1:** Minimal Inhibitory Concentration (MIC) determinations of the selected compounds against *Cryptococcus* sp., *C. auris*, and *C. haemulonii*.

		PH265		PH276		Amphotericin B *		Fluconazole		Caspofungin	
Isolate		µM	µg/mL	µM	µg/mL	µM	µg/mL	µM	µg/mL	µM	µg/mL
*Cryptococcus neoformans*	H99	3	1	1.5	0.5	0.54	0.5	208.9	8	-	-
*Cryptococcus deneoformans*	B3501	1.5	0.5	12.38	4	0.135	0.125	6.525	2	-	-
*Cryptococcus deuterogattii*	R265	3	1	1.5	0.5	0.27	0.25	26.12	8	-	-
*Candida haemulonii*	LipCh12	3	1	24.76	8	2.165	2	26.12	8	3.65	4
*Candida auris*	Ca446	3	1	24.76	8	0.54	0.5	26.12	8	1.82	2

* 90% inhibition.

**Table 2:** Minimal Fungicidal Concentration (MFC) determinations of the selected compounds against *Cryptococcus* spp., *C. auris*, and *C. haemulonii*.

		PH265		PH276		Amphotericin B		Fluconazole		Caspofungin	
Isolate		µM	µg/mL	µM	µg/mL	µM	µg/mL	µM	µg/mL	µM	µg/mL
*Cryptococcus neoformans*	H99	24.6	8	6.19	2	0.54	0.5	208.9	64	-	-
*Cryptococcus deneoformans*	B3501	>49.5	>16	49.5	16	0.54	0.5	104.4	32	-	-
*Cryptococcus deuterogattii*	R265	>49.5	>16	6.19	2	0.54	0.5	208.9	>64	-	-
*Candida haemulonii*	LipCh12	>49.5	>16	>49.5	>16	2.165	2	208.9	>64	7.31	8
*Candida auris*	Ca446	>49.5	>16	>49.5	>16	0.54	0.5	208.9	>64	>32	>29.26

**Table 3:** Impact of the association of PH 265 and PH 276 (drug A) with amphotericin B (AmB), fluconazole (Flu), or caspofungin (Caspo) (drug B) on the antifungal activity against *C. neoformans*, *C. auris*, and *C. haemulonii*.

	Drug A		Drug B			Drug A	
Strain	Molecule	MIC Alone (µg/mL)	MIC Combined (µg/mL)		MIC Alone (µg/mL)	MIC Combined (µg/mL)	FIC ^1^
*C. neoformans* H99	PH265	8	1	FLZ	8	8	1.125
	PH265	1	1	AmB	0.5	0.5	1
	PH276	0.5	0.0312	FLZ	8	8	1.06
	PH276	0.5	0.5	AmB	0.5	0.5	1
*C. auris* Ca446	PH265	1	0.125	FLZ	8	8	1.12
	PH265	1	1	AmB	0.5	0.06	1.12
	PH265	1	1	Caspo	1	1	1
	PH276	8	0.5	FLZ	8	8	1
	PH276	8	8	AmB	0.5	0.5	1
	PH276	8	8	Caspo	1	0.5	1.5
*C. haemulonii* LipCh12	PH265	1	1	FLZ	8	4	1.5
	PH265	1	1	AmB	2	2	1
	PH265	1	0.5	Caspo	4	0.5	0.75
	PH276	8	1	FLZ	8	2	0.37
	PH276	8	8	AmB	2	0.06	1
	PH276	8	2	Caspo	4	1	0.5

^1^ Fractional inhibitory concentration index (FIC); synergistic: FIC ≤ 0.5; indifferent FIC > 0.5–4; antagonist: FIC > 4.

## Data Availability

Data are contained within the article required.

## Abbreviations

The following abbreviations are used in this manuscript:

8HQ	8-hydroxyquinoline
MIC	Minimal Inhibitory Concentration
MFC	Minimal Fungicidal Concentration

## References

[1] Denning D.W., Bromley M.J. (2015). Infectious Disease. How to Bolster the Antifungal Pipeline. Science.

[2] Rodrigues M.L., Nosanchuk J.D. (2020). Fungal Diseases as Neglected Pathogens: A Wake-up Call to Public Health Officials. PLoS Negl. Trop. Dis..

[3] Ademe M., Girma F. (2020). *Candida auris*: From Multidrug Resistance to Pan-Resistant Strains. Infect. Drug Resist..

[4] Chowdhary A., Voss A., Meis J.F. (2016). Multidrug-Resistant *Candida auris*: “new Kid on the Block” in Hospital-Associated Infections?. J. Hosp. Infect..

[5] Du H., Bing J., Hu T., Ennis C.L., Nobile C.J., Huang G. (2020). *Candida auris*: Epidemiology, Biology, Antifungal Resistance, and Virulence. PLoS Pathog..

[6] Arora P., Singh P., Wang Y., Yadav A., Pawar K., Singh A., Padmavati G., Xu J., Chowdhary A. (2021). Environmental Isolation of *Candida auris* from the Coastal Wetlands of Andaman Islands, India. mBio.

[7] Bongomin F., Oladele R.O., Gago S., Moore C.B., Richardson M.D. (2018). A Systematic Review of Fluconazole Resistance in Clinical Isolates of *Cryptococcus* Species. Mycoses.

[8] Naicker S.D., Mpembe R.S., Maphanga T.G., Zulu T.G., Desanto D., Wadula J., Mvelase N., Maluleka C., Reddy K., Dawood H. (2020). Decreasing Fluconazole Susceptibility of Clinical South African *Cryptococcus* neoformans Isolates over a Decade. PLoS Negl. Trop. Dis..

[9] Rajasingham R., Smith R.M., Park B.J., Jarvis J.N., Govender N.P., Chiller T.M., Denning D.W., Loyse A., Boulware D.R. (2017). Global Burden of Disease of HIV-Associated Cryptococcal Meningitis: An Updated Analysis. Lancet Infect. Dis..

[10] Martinez L.R., Casadevall A. (2015). Biofilm Formation by *Cryptococcus neoformans*. Microbiol. Spectr..

[11] Krysan D.J. (2015). Toward Improved Anti-Cryptococcal Drugs: Novel Molecules and Repurposed Drugs. Fungal Genet. Biol..

[12] Rodrigues M.L. (2016). Funding and Innovation in Diseases of Neglected Populations: The Paradox of Cryptococcal Meningitis. PLoS Negl. Trop. Dis..

[13] Sloan D.J., Dedicoat M.J., Lalloo D.G. (2009). Treatment of Cryptococcal Meningitis in Resource Limited Settings. Curr. Opin. Infect. Dis..

[14] Berkow E.L., Lockhart S.R. (2017). Fluconazole Resistance in *Candida* Species: A Current Perspective. Infect. Drug Resist..

[15] Mpoza E., Rhein J., Abassi M. (2018). Emerging Fluconazole Resistance: Implications for the Management of Cryptococcal Meningitis. Med. Mycol. Case Rep..

[16] de Oliveira H.C., Castelli R.F., Alves L.R., Nosanchuk J.D., Salama E.A., Seleem M., Rodrigues M.L. (2022). Identification of Four Compounds from the Pharmakon Library with Antifungal Activity against *Candida auris* and Species of Cryptococcus. Med. Mycol..

[17] Denning D.W. (2003). Echinocandin Antifungal Drugs. Lancet.

[18] Chan-On W., Huyen N.T.B., Songtawee N., Suwanjang W., Prachayasittikul S., Prachayasittikul V. (2015). Quinoline-Based Clioquinol and Nitroxoline Exhibit Anticancer Activity Inducing FoxM1 Inhibition in Cholangiocarcinoma Cells. Drug Des. Dev. Ther..

[19] Lazovic J., Guo L., Nakashima J., Mirsadraei L., Yong W., Kim H.J., Ellingson B., Wu H., Pope W.B. (2015). Nitroxoline Induces Apoptosis and Slows Glioma Growth in Vivo. Neuro Oncol..

[20] Naber K.G., Niggemann H., Stein G., Stein G. (2014). Review of the Literature and Individual Patients’ Data Meta-Analysis on Efficacy and Tolerance of Nitroxoline in the Treatment of Uncomplicated Urinary Tract Infections. BMC Infect. Dis..

[21] Prachayasittikul V., Prachayasittikul S., Ruchirawat S., Prachayasittikul V. (2013). 8-Hydroxyquinolines: A Review of Their Metal Chelating Properties and Medicinal Applications. Drug Des. Dev. Ther..

[22] Cherdtrakulkiat R., Boonpangrak S., Sinthupoom N., Prachayasittikul S., Ruchirawat S., Prachayasittikul V. (2016). Derivatives (Halogen, Nitro and Amino) of 8-Hydroxyquinoline with Highly Potent Antimicrobial and Antioxidant Activities. Biochem. Biophys. Rep..

[23] Pippi B., Loreto E.S., Merkel S., Joaquim A.R., Krummenauer M.E., Reginatto P., Vainstein M.H., Andrade S.F., Fuentefria A.M., Santurio J.M. (2023). *Pythium insidiosum*: Insights into Biofilm Formation and Antibiofilm Activity of Antifungal Drugs. Braz. J. Microbiol..

[24] Joaquim A.R., Boff R.T., Adam F.C., Lima-Morales D., Cesare M.A., Kaminski T.F., Teixeira M.L., Fuentefria A.M., Andrade S.F., Martins A.F. (2022). Antibacterial and Synergistic Activity of a New 8-Hydroxyquinoline Derivative against Methicillin-Resistant *Staphylococcus aureus*. Future Microbiol..

[25] Pippi B., Zanette R.A., Joaquim A.R., Krummenauer M.E., Merkel S., Reginatto P., Vainstein M.H., Andrade S.F., Fuentefria A.M., Tondolo J.S.M. (2022). Clioquinol and 8-Hydroxyquinoline-5-Sulfonamide Derivatives Damage the Cell Wall of *Pythium insidiosum*. J. Appl. Microbiol..

[26] Kadri D., Crater A.K., Lee H., Solomon V.R., Ananvoranich S. (2014). The Potential of Quinoline Derivatives for the Treatment of *Toxoplasma gondii* Infection. Exp. Parasitol..

[27] da Silva N.M., Gentz C. (2021). de B.; Reginatto, P.; Fernandes, T.H.M.; Kaminski, T.F.A.; Lopes, W.; Quatrin, P.M.; Vainstein, M.H.; Abegg, M.A.; Lopes, M.S.; et al. 8-Hydroxyquinoline 1,2,3-Triazole Derivatives with Promising and Selective Antifungal Activity. Med. Mycol..

[28] Gentz C.d.B., Lopes M.S., Quatrin P.M., Gionbelli M.P., de Cesare M.A., Perin A.P., Lopes W., Fuentefria A.M., Vainstein M.H., Andrade S.F.d. (2025). A Potent Fluorescent Derivative of 8-Hydroxyquinoline Suggests Cell Wall Damage as a Possible Cellular Action of the 5-Triazole 8-Hydroxyquinoline Class. Appl. Microbiol..

[29] Brenner S. (1974). The Genetics of Caenorhabditis elegans. Genetics.

[30] Porta-de-la-Riva M., Fontrodona L., Villanueva A., Cerón J. (2012). Basic *Caenorhabditis elegans* Methods: Synchronization and Observation. J. Vis. Exp..

[31] Martinez L.R., Ntiamoah P., Gácser A., Casadevall A., Nosanchuk J.D. (2007). Voriconazole Inhibits Melanization in *Cryptococcus neoformans*. Antimicrob. Agents Chemother..

[32] Reis F.C.G., Borges B.S., Jozefowicz L.J., Sena B.A.G., Garcia A.W.A., Medeiros L.C., Martins S.T., Honorato L., Schrank A., Vainstein M.H. (2019). A Novel Protocol for the Isolation of Fungal Extracellular Vesicles Reveals the Participation of a Putative Scramblase in Polysaccharide Export and Capsule Construction in *Cryptococcus gattii*. mSphere.

[33] Kwon-Chung K.J., Wickes B.L., Booth J.L., Vishniac H.S., Bennett J.E. (1987). Urease Inhibition by EDTA in the Two Varieties of *Cryptococcus neoformans*. Infect. Immun..

[34] Lai Y.-W., Campbell L.T., Wilkins M.R., Pang C.N.I., Chen S., Carter D.A. (2016). Synergy and Antagonism between Iron Chelators and Antifungal Drugs in *Cryptococcus*. Int. J. Antimicrob. Agents.

[35] Joffe L.S., Schneider R., Lopes W., Azevedo R., Staats C.C., Kmetzsch L., Schrank A., Del Poeta M., Vainstein M.H., Rodrigues M.L. (2017). The Anti-Helminthic Compound Mebendazole Has Multiple Antifungal Effects against *Cryptococcus neoformans*. Front. Microbiol..

[36] Ribeiro N.S., Dos Santos F.M., Garcia A.W.A., Ferrareze P.A.G., Fabres L.F., Schrank A., Kmetzsch L., Rott M.B., Vainstein M.H., Staats C.C. (2017). Modulation of Zinc Homeostasis in *Acanthamoeba castellanii* as a Possible Antifungal Strategy against *Cryptococcus gattii*. Front. Microbiol..

[37] Silva M.G., de Curcio J.S., Silva-Bailão M.G., Lima R.M., Tomazett M.V., de Souza A.F., Cruz-Leite V.R.M., Sbaraini N., Bailão A.M., Rodrigues F. (2020). Molecular Characterization of Siderophore Biosynthesis in *Paracoccidioides brasiliensis*. IMA Fungus.

[38] dos Santos Ramos F., Martins D.M., Nunes Sagini J.P., Quines C.B., de Oliveira Pereira F.S., de Ávila D.S., Zanzarin D., Pilau E.J., de Lima Postiga I.A., Tostes J. (2021). Soybeans Agroindustrial Residues as *Staphylococcus epidermidis* and *S. aureus* Biofilm Inhibitors. Ind. Crops Prod..

[39] Vanin A.P., Tamagno W.A., Alves C., Mesacasa L., Santin L.F., Sutorillo N.T., Bilibio D., Müller C., Galon L., Kaizer R.R. (2022). Neuroprotective Potential of *Cannabis sativa*-Based Oils in *Caenorhabditis elegans*. Sci. Rep..

[40] Limited Availability of Generic Antifungals Across the World. https://gaffi.org/limited-availability-of-generic-antifungals-across-the-world/41.

[41] Deray G. (2002). Amphotericin B Nephrotoxicity. J. Antimicrob. Chemother..

[42] Meis J.F., Chowdhary A. (2019). *Candida auris*-“Ten Years After”. J. Fungi.

[43] Joaquim A.R., Pippi B., de Cesare M.A., Rocha D.A., Boff R.T., Staudt K.J., Ruaro T.C., Zimmer A.R., de Araújo B.V., Silveira G.P. (2018). Rapid Tools to Gain Insights into the Interaction Dynamics of New 8-Hydroxyquinolines with Few Fungal Lines. Chem. Biol. Drug Des..

[44] Martínez-Ballesta M., Gil-Izquierdo Á., García-Viguera C., Domínguez-Perles R. (2018). Nanoparticles and Controlled Delivery for Bioactive Compounds: Outlining Challenges for New “Smart-Foods” for Health. Foods.

[45] Nosanchuk J.D., Ovalle R., Casadevall A. (2001). Glyphosate Inhibits Melanization of *Cryptococcus neoformans* and Prolongs Survival of Mice after Systemic Infection. J. Infect. Dis..

